# The Benefits of Adding Sulfur and Urea to a Concentrate Mixture on the Utilization of Feed, Rumen Fermentation, and Milk Production in Dairy Cows Supplemental Fresh Cassava Root

**DOI:** 10.1155/2022/9752400

**Published:** 2022-10-03

**Authors:** Phussorn Sumadong, Sarong So, Anusorn Cherdthong

**Affiliations:** ^1^Tropical Feed Resources Research and Development Center (TROFREC), Department of Animal Science, Faculty of Agriculture, Khon Kaen University, Khon Kaen 40002, Thailand; ^2^Department of Animal Science, Faculty of Agriculture and Food Processing, National University of Battambang, Battambang 02352, Cambodia

## Abstract

Fresh cassava roots that contain hydrocyanic acid (HCN) can be hazardous to animals when consumed. Prior literature has shown that adding sulfur may eliminate HCN without harming the health of animals. Additionally, adding urea is advised if sulfur was utilized since it helps with microbial protein synthesis. We thus proposed that supplementing the fresh cassava root diet with a high sulfur and urea in concentrate diet would be advantageous for rumen fermentation and milk production in animals. The purpose of this study was to see how high sulfur and urea levels in concentrate combinations affected feed utilization, rumen fermentation, and milk production in dairy cows fed diets including fresh cassava root. Four Holstein Friesian cows with 480 ± 50.0 kg BW, 10 ± 2 kg/head/day of milk yield, and 90 days in milk (DIM) were assigned at random in a 4 × 4 Latin square design with a 2 × 2 factorial design. Factor A was the concentration of sulfur in the concentrate diet at 10 g/kg and 20 g/kg dry matter (DM), while factor B was the concentration of urea in the concentrate diet at 10 g/kg and 20 g/kg DM. Fresh cassava root was given to each cow on a daily basis at a rate of 15 g DM/kg of BW. According to the findings, sulfur and urea had no interaction impact on feed intake, rumen fermentation, or milk production. Sulfur supplementation at 20 g/kg DM improved sulfur intake and digestibility of DM and organic matter much more than 10 g/kg sulfur. Additionally, sulfur supplementation at a dose of 20 g/kg DM in concentrate markedly increased blood and milk thiocyanate concentrations while lowering the somatic cell count. When compared to 10 g/kg DM urea, 20 g/kg DM urea significantly enhanced crude protein digestibility, ammonia-nitrogen concentration, blood urea nitrogen, and total volatile fatty acid concentration. Sulfur might detoxify hydrogen cyanide toxicity and be added at 20 g/kg DM in concentrate without harming the animals, whereas urea at 20 g/kg DM could increase feed digestion and rumen fermentation.

## 1. Background

Farmers are still concerned about the presence of hydrocyanic acid (HCN) in fresh cassava root when employing fresh cassava root as an energy source in ruminant diets. Consuming feed that contains 500 mg of HCN/kg of dry weight may be detrimental to the host [1]. On the other hand, sun-drying may reduce HCN, and sun-dried cassava roots are extensively utilized as an energy source in ruminant diets [2, 3]. Although it takes time and is ineffective during the rainy season, sun-drying is a simple process for eliminating HCN from fresh cassava root.

A previous study revealed that adding sulfur might help to reduce HCN toxicity in fresh cassava root [4–6]. Cherdthong et al. [7] found that adding 40 g/kg sulfur to mineral blocks and supplementing 15 g/kg DM of fresh cassava root to the diet increased blood thiocyanate while having no negative effects on rumen fermentation, feed intake, or blood urea nitrogen. Supapong et al. [8] discovered that adding 20 g/kg sulfur to a fermented total mixed ration (FTMR) that contained fresh cassava root increased the blood levels of thiocyanate, digestibility, and synthesis of microbial protein. The process of adding sulfur to manufacture mineral blocks, or FTMR, was complicated and might not be practical for farmers [9]. Feed blocks are needed to be formed by a machine and allowed time to make a dry form. Additionally, the consumption of sulfur was dependent on animal licks, which may mean that there was not enough sulfur to minimize the HCN. Sulfur inclusion in FTMR requires a TMR machine to chop the feeds and mix the TMR, and when the TMR is fermented, it requires plastic bags or a silo for anaerobic fermentation. Furthermore, the fermentation process of FTMR required at least 7 to 14 days before feeding, whereas adding sulfur to concentrate allowed for feeding after. This suggests that since the practical technique is more applicable than past research and fills a research gap in the current study, it should be taken into consideration [3]. The addition of sulfur to concentrate diets would be a reasonable method to address the research gap and provide more immediate relief for the abovementioned issue.

Urea is a common nonprotein nitrogen (NPN) source that is utilized in ruminant nutrition to boost the protein content of feed items and as a nitrogen source in the rumen to synthesize microbial protein [10]. Improvements in microbial protein synthesis and utilization may result from a balance in the daily ratio of rumen with urea to available carbohydrates in the feed [11]. Recently, Cherdthong et al. [7] showed that concentrates based on a high proportion of fermentable carbohydrate sources with a high amount of urea might increase the efficiency of ruminal fermentation and the ruminal synthesis of microbial CP in dairy cows. Moreover, the metabolism of nitrogen and sulfur is closely linked. There is a need to clarify the ideal level of sulfur supplementation in urea-containing diets [9]. The animal depends on rumen microbes to convert sulfate into hydrogen sulfite, which is then utilized to produce methionine and cysteine for microbial growth [8]. Thus, the constant availability of urea and sulfur in the fermentable carbohydrate source given to animals may enhance microbial populations and improve DM digestibility [7]. An earlier study found that adding urea and sulfur to feed blocks or FTMR improved the dry matter digestibility, bacterial populations, propionic acid concentrations, and milk composition in animals fed fresh cassava root [7, 9].

We thus hypothesized that it would be beneficial for rumen fermentation and milk production in animals to supplement the fresh cassava root diet with a high sulfur and urea in concentrate diet. Therefore, the objective of the study was to investigate the effects of sulfur and urea addition to concentrate mixes on the utilization of feed, rumen fermentation, and milk production in dairy cows given fresh cassava root-containing diets.

## 2. Materials and Methods

### 2.1. Animal Ethical Approval

The usage and care of cows were carried out in accordance with Khon Kaen University's ethical permission number: ACUC-KKU 32/61.

### 2.2. Cows, Study Design, and Diet

Due to the limited number of cows at the farm-research station, this study was done at the Department of Animal Science, Khon Kaen University, with a small number of cows. Incidental mistakes caused by humans, cows, and the environment, on the other hand, were strictly managed. Four Holstein Friesian cows with 480 ± 50.0 kg BW and 90 days in milk (DIM) were assigned at random in a 4 × 4 Latin square design with a 2 × 2 factorial design. Factor A was the concentration of sulfur in the concentrate diet at 10 g/kg and 20 g/kg dry matter (DM), while factor B was the concentration of urea in the concentrate diet at 10 g/kg and 20 g/kg DM. The four treatment combinations were as follows: (1) 10 g/kg DM of sulfur + 10 g/kg DM of urea; (2) 10 g/kg DM of sulfur + 20 g/kg DM of urea; (3) 20 g/kg DM of sulfur + 10 g/kg DM of urea; and (4) 20 g/kg DM of sulfur + 20 g/kg DM of urea, respectively. In Table 1, the ingredients of the concentrate diet are listed. The cows were housed in 5 m^2^ separate pens with lots of clean water and mineral blocks.

### 2.3. Feeding Procedure

This study was divided into four periods, each of which lasted 14 days for treatment adjustment and 7 days for sample collection. A concentrated combination of sulfur and urea was fed to cows twice a day, at 7:00 a.m. and 4:00 p.m., in a 2 : 1 milk yield ratio. The cows were given rice straw (RS) as their main roughage source ad libitum, with a refusal allowance of 100 g/kg DM. A one-year-old fresh cassava root (Kasetsart 50, bitter variety) was obtained from a local farmer in Khon Kaen province, cleaned, and chopped by machine into 0.3 to 0.5 cm pieces. Rice straw, fresh cassava root, and concentrated feed were all given individually. Daily top-up supplementation of fresh cassava root at a level of 15 g DM/kg of BW was given.

### 2.4. Sample Collection and Analysis

The intake of rice straw, concentrate, and fresh cassava root, as well as their refusals, was recorded daily throughout the trial in order to compute the daily intake of dry matter (DM) expressed in kilograms per day. During the last 7 days of each period, the feeds presented, as well as their refusal and feces were tested in the morning and afternoon feeding. The rectal sampling technique was used to obtain fecal samples. The feed, refuse, and feces samples were divided into two parts: each sample was divided into two parts: the first part was daily processed for DM content, and the second part was combined by cows and periods and stored at −20°C for further analysis. The samples were taken from storage and thawed at room temperature in order to assess their chemical composition. The thawed samples of feeds, refusal, and 50 g/kg feces on fresh matter from total feces were then oven-dried for 72 hours at 60°C and ground through a 1 mm screen plate. According to AOAC [12], ground samples of feeds, refusals, and feces were analyzed for chemical contents such as DM (ID 967.03), organic matter (OM, ID 942.05), crude protein (CP, ID 976.05), and neutral detergent fiber (NDF) and acid detergent fiber (ADF) using a fiber analyzer (ANKOM 200, ANKOM Technology, New York, USA) [13]. The digestibility coefficient was estimated using the acid insoluble ash approach (AIA) [14]. The concentration of hydrogen cyanide (HCN) in fresh cassava root was determined using the Bradbury et al. [15] technique. The HCN concentration was given in milligrams per kilogram.

On day 21 of each period, rumen fluid and blood samples were obtained at 0 and 4 hours after feeding. A stomach tube connected to a vacuum machine was used to collect 100 ml of whole rumen fluid. The pH and temperature of the rumen were measured immediately after collection using a glass electrode pH meter (HANNA Instrument HI 8424 microcomputer, Singapore). The rumen fluid was then filtered through four layers of cheesecloth and stored in 1 M sulfuric acid at a 1 : 9 ratio (5 ml H_2_SO_4_ and 45 ml rumen fluid). The filtered rumen fluid was centrifuged at 16,000 g for 15 minutes to determine the concentrations of ammonia-nitrogen (NH_3_-N) and volatile fatty acids (VFAs). The NH_3_-N content was determined using the AOAC technique [12], and the VFAs concentration, which included acetate (C2), propionate (C3), and butyrate (C4), was determined by high-performance liquid chromatography (HPLC; Model Water 600; UV detector, Millipore Crop). The remaining rumen fluid was combined with 10% formalin in a 1 : 9 ratio (1 ml rumen fluid and 9 ml formalin) and utilized for a direct count of protozoa using a hemocytometer. Ten milliliters of blood were drawn from a jugular vein and split into two parts: the first 5 ml of blood was maintained in EDTA tubes and used to evaluate blood urea nitrogen (BUN) according to Cherdthong et al. [7], and another 5 ml of blood was stored in tubes and centrifuged immediately to extract serum and used to assess blood thiocyanate according to Lambert et al. [16]. According to Lambert et al. [16], plasma samples were tested for alanine aminotransferase (ALT), aspartate aminotransferase (AST), triiodothyronine (T3), and thyroxine (T4) using automated clinical chemistry analyzers (Vitallab Flexor E).

Each cow's milk output was reported every day. A 100 ml milk sample was collected daily by combining 60 ml of milk from morning milking at 5:00 a.m. and 40 ml of milk from afternoon milking at 4:00 p.m., and it was sent to the laboratory to be analyzed for fat, protein, lactose, solids-not-fat, total solids, and milk urea nitrogen content using an infrared apparatus (Milkoscan104, Foss Electric, Denmark). The somatic cell count in milk samples was determined using the Fossomatic 5000 Basic. According to Jacob et al. [17], the thiocyanate content (SCN^−^) in milk samples was determined.

## 3. Statistical Methods

The data were analyzed using the general linear procedure in SAS procedures in a 2 × 2 factorial arrangement in a 4 × 4 Latin square design (SAS Inst. Inc., Cary, NC). The statistical model terms include the concentrations of sulfur and urea in the concentrate, as well as their interactions. Duncan's new multiple range tests (*P* < 0.05) were used to evaluate differences in treatment means.

## 4. Results

### 4.1. Dietary Nutritive Value

The nutritive values of concentrates, fresh cassava root, and RS are shown in Table 1. Cassava chip and fresh cassava root are the primary energy sources in the diet, whereas RS is the primary roughage source. To achieve the requirement, a concentrate containing 162.0 to 186.4 g/kg DM was generated in accordance with NRC [4]. 0.105 g HCN/kg DM was found in the fresh cassava root (Table 1).

### 4.2. Intake and Nutrient Digestibility

Table 2 shows the effect of sulfur and urea on feed intake and nutrient digestibility. No interaction between sulfur and urea affected how well nutrients were ingested or assimilated. The sulfur addition had no effect on RS, concentrate, fresh cassava root, HCN, or total DM consumption, but it had a significant effect on sulfur intake. When the amount of sulfur in the feed increased, thus did the amount of sulfur consumed. The sulfur consumption between sulfur-1 and sulfur-2 was 56.25 g/d and 112.5 g/d, respectively. The addition of urea had no effect on feed consumption. Sulfur had a significant effect on DM and OM digestibility but had no effect on CP, NDF, or ADF digestibility. The DM and OM digestibility of sulfur-1 and sulfur-2 were 657.2 g/kg DM and 685.7 g/kg DM, respectively, and 699.4 g/kg DM and 714.0 g/kg DM, respectively. The addition of urea has a significant impact on CP digestibility.

### 4.3. Ruminal pH, Ammonia-Nitrogen, Protozoa, and Bacteria Population

The ruminal pH, NH_3_-N concentration, and protozoa and bacterial populations responded to sulfur and urea addition are shown in Table 3. There was no interactive effect between sulfur and urea on pH, NH_3_-N or protozoa except that the bacteria population at 4 h after feeding was affected by urea and sulfur addition. The addition of sulfur had no effect on pH, NH_3_-N, protozoa, or bacteria. Urea had a significant effect on NH_3_-N concentration but had no effect on pH, protozoa, or bacteria. Urea-1 and urea-2 had average NH_3_-N concentrations of 13.82 mg/dl and 17.49 mg/dl, respectively.

### 4.4. Serum Thiocyanate, Blood Urea Nitrogen, Thyroid Hormones, and Liver Enzymes

Table 4 shows the effects of sulfur and urea additions on blood SCN^−^ concentrations, BUN, thyroid hormones, and liver enzymes. There was no interaction between sulfur and urea in the serum of SCN^−^, BUN, T3, T4, ALT, or AST. Sulfur had a significant effect on serum SCN^−^ concentration but had no effect on BUN, thyroid hormones, or liver enzymes. Serum SCN^−^ levels were 13.5 g/ml for sulfur-1 and 16.12 g/ml for sulfur-2, respectively. Urea had a significant effect on BUN concentration but had no effect on serum SCN^−^, thyroid hormones, or liver enzymes. Urea-1 and urea-2 had BUN concentrations of 8.89 mg/dl and 9.59 mg/dl, respectively.

### 4.5. Volatile Fatty Acid Production

Table 5 shows the VFA, C2, C3, and C4 concentrations. Total VFA, C2, C3, and C4 concentrations were not affected by sulfur or urea. The addition of sulfur had no effect on the synthesis of C2, C3, and C4, as well as the overall VFA content. Urea had a significant effect on overall VFA concentration after 4 hours of feeding but had no effect on the molar components of VFA such as C2, C3, and C4. After 4 hours of feeding, the total VFA concentrations in urea-1 and urea-2 were 117.6 mM and 130.15 mM, respectively.

### 4.6. Milk Yield and Its Composition

The effects of sulfur and urea on milk production, milk composition, milk SCN^−^, and milk SCC are shown in Table 6. There was no interaction between sulfur and urea on milk output, 3.5% FCM, fat, protein, lactose, solid-not-fat, total solid, milk SCN^−^, and SCC. Sulfur had a significant effect on milk SCN^−^ and SCC but had no effect on milk output or composition. Milk SCN^−^ and SCC levels were 7.34 ppm and 8.64 ppm, respectively, and 297.81 × 10^3^ cell/ml and 229.0 × 10^3^ cell/ml, respectively.

## 5. Discussion

The HCN concentration of the fresh cassava root employed in this investigation was 6% lower than that reported by [8]. Morgan and Choct [1] observed that the HCN level ranged from 0.015 to 0.400 g HCN/kg of fresh materials. HCN content can vary according to a variety of factors such as variety, terrain, seasons, and geographical places [8].

Sulfur accumulates at a rate of 1.5 g/kg DM. According to NRC [4], animals require 2 g/kg of sulfur in dietary DM to support optimal microbial growth and microbial protein synthesis with a maximum tolerance level of 4 g/kg of sulfur. Sulfur is vital for rhodanese enzymes in converting HCN into a less hazardous thiocyanate when an animal consumes meals containing HCN sources, in addition to being useful for microbial growth and microbial protein synthesis. Raised sulfur consumption was dramatically increased by increasing sulfur in the diet. Sulfur consumption as a percentage of total DM intake was 0.35% for sulfur-1 and 0.69% for sulfur-2. Although the sulfur intake expressed as a percentage of total DM intake was higher than the NRC's recommended maximum tolerance level [4], the animal showed no negative symptoms, and this amount of sulfur may be beneficial for both microbial growth and rhodanese enzyme activity in converting HCN to less toxic thiocyanate. According to Cherdthong et al. [7], cattle consumed 0.53% of their total DM intake when 40 g/kg of sulfur was added to the feed block. Prachumchai et al. [6] found that the ingestion of sulfur accounted for 1.08 percent of total DM intake when subjects were given a pellet containing 30 g of DM's sulfur content. Daily urea consumption rose significantly as concentrate's urea content increased. This may be attributed to the rise in the amount of urea in concentrate. The addition of urea had no effect on feed consumption. In this investigation, cows given 20 g/kg of DM concentrate urea-containing concentrate had a maximum urea consumption of 112.50 g/d. Supapong and Cherdthong. [9] discovered that when 25 g/kg DM urea-containing diets were provided without influencing feed intake, cows consumed around 371 g/d; this might be explained by the synchronization of urea and energy as diets had high fresh cassava root. High digestible carbohydrate components such as cassava chips or fresh cassava root may improve urea utilization in the rumen, resulting in minimal urea toxicity or influence on feed intake.

Sulfur is required for the synthesis of amino acids and vitamins in the rumen, including methionine, cysteine, B-vitamins, thiamin, and biotin, as well as microbial proliferation [4]. The increase in DM and OM digestibility at 20 g/kg DM sulfur in the concentrate showed that sulfur might boost microbial activity in the rumen to decompose nutrients. Promkot et al. [3] found that adding 4 g/kg of sulfur to the diet improved fiber digestibility. Supapong et al. [8] also found that adding 20 g/kg of sulfur to FTMR increased DM digestibility. According to Cherdthong et al. [7], increasing the feed block's sulfur content by 40 g/kg increased the digestibility of DM and OM. The digestibility of CP was increased by adding 20 g/kg urea. The increase in urea breakdown in the rumen may be explained by the synchronization with fresh cassava root administration. The combination of nitrogen and energy is essential for microbial protein synthesis [3, 9]. According to Khampa et al. [18], a diet high in urea might be helpful when combined with a highly fermentable carbohydrate such as cassava chips. According to Getahun et al. [19], adding extra energy sources like grain, starch, or dried beet pulp can significantly speed up the rumen's breakdown of urea. Similar findings were made by Emmanuel et al. [20], who found that feeding growing camels full pellet diets based on roughage significantly improved CP digestibility at 10 and 20 g/kg compared to the control. Using urea-CaCl_2_ and urea-CaSO_4_ at 6.7 percent in a diet high in cassava chips did not impact CP digestibility when compared to the control, according to Cherdthong et al. [10]. This could be because urea-CaCl_2_ and urea-CaSO_4_ products have properties that allow for delayed release in the rumen.

The addition of sulfur had no effect on ruminal pH, NH_3_-N, protozoa, or bacteria. The pH of the rumen varied from 6.6 to 6.5. The pH was within the range required for microbial activity [10]. Ruminal pH that is too high or too low may have an influence on ruminal microbial proliferation and fermentation. Rumsey [21] reported that high sulfur diets reduce ruminal pH in ruminants after 2 hours of eating, although the pH steadily increases afterward [22]. According to Deng et al. [22], the time gap between feeding sulfur-containing diets affects ruminal pH. Morine et al. [23] discovered that, after 6 hours of feeding, H_2_S generation was inversely linked with pH. Increased ruminal pH may reduce H_2_S generation by suppressing the action of sulfate-reducing bacteria. Deng et al. [22] found that using different sulfate sources had little effect on ruminal pH. Cherdthong et al. [7] employed 40 g/kg sulfur in the feed block and found that it had no influence on the pH. Supapong and Cherdthong [9] demonstrated that 20 g/kg sulfur addition had no effect on ruminal pH. When 10 and 20 g/kg of sulfur were added, the ruminal NH_3_-N concentration varied from 15.35 to 15.97 mg/dl (Table 3). The level of NH_3_-N was more than adequate for the synthesis of microbial proteins. Bacteria can synthesize ruminal microbial protein by utilizing NH_3_-N and carbohydrates for protein synthesis as well as sulfur for the synthesis of sulfur-containing amino acids [3]. This might explain why the NH_3_-N concentrations with sulfur addition at 10 g/kg and 20 g/kg were nonsignificant since sulfur was used independently for microbial growth, protein synthesis, and HCN detoxification. The NH_3_-N level increased after 4 hours of feeding sulfur-containing meals, which might be attributed to a high rate of urea breakdown in the rumen. Deng et al. [22] discovered a decrease in NH_3_-N 4 hours after feeding meals containing diverse sulfur sources; this might be explained by the low breakdown of protein received from the diets in the rumen or supplied without external nonprotein nitrogen addition. Cherdthong et al. [7] discovered that 40 g/kg sulfur in the feed block had no influence on NH_3_-N. Supapong and Cherdthong [9] found that adding 20 g/kg of sulfur to FTMR diets had no effect on NH_3_-N content. The bacterial population was not significantly different between 10 and 20 g/kg sulfur. This revealed that including up to 20 g/kg of sulfur in the diet had no effect on the bacterial population, but it is sufficient for microbial growth and activity, as well as HCN detoxification. According to Promkot et al. [3], sulfur supplementation might be beneficial when the diet includes less sulfur than the NRC recommendation of a 1 g/kg diet [4]. The NH_3_-N content was dramatically increased with the addition of 20 g/kg urea. The rise in NH_3_-N content after urea addition indicated that urea was extensively digested in the rumen. According to Hristov et al. [24], raising CP digestibility led to a rise in NH_3_-N concentration; Table 2 amply demonstrates this. These data corroborated those of Supapong and Cherdthong [9], who found that 25 g/kg urea caused a considerable increase in NH_3_-N concentration. At 160 g/kg DM CP as opposed to 140 g/kg DM CP in the diets, Unnawong et al.'s [25] research revealed that the NH_3_-N content significantly increased.

At 20 g/kg sulfur addition, serum SCN^−^ rose considerably. This might be explained by the activity of the rumen-produced rhodanese enzyme, which accepts sulfur and converts HCN into thiocyanate, which then enters the circulation [3]. Then, thiocyanate interacts with hemoglobin to form cyanohemoglobin, a nonoxygen carrier; hence, diet HCN increased the quantity of serum SCN^−^ in urine and blood. According to Sousa et al. [26], serum thiocyanate has no effect on thyroid hormone levels. Furthermore, a rise in serum SCN^−^ concentration may result from an increase in HCN and sulfur consumption. Prachumchai et al. [6] added 15 and 30 g/kg sulfur in pellets, Cherdthong et al. [7] added 40 g/kg sulfur to the feed block, and Supapong and Cherdthong [9] added 20 g/kg sulfur to FTMR diets. The results of Cherdthong et al. [7] who observed that adding sulfur to meals at 4 g/kg DM had no effect on T3, T4, ALT, and ASTS were supported by the finding that sulfur had no effect on thyroid hormones (T3 and T4) or liver enzymes (ALT and ASTS). Due to an increase in CP digestibility and NH_3_-N concentration, BUN content significantly increased after 20 g/kg urea treatment. According to Sarwar et al. [27], BUN displayed a positive relationship with NH_3_-N concentration. Numerous earlier studies have discovered a significant increase in BUN concentration, including Unnawong et al. [25], who increased the CP level from 140 to 160 g/kg in concentrate fed to beef cattle, Supapong and Cherdthong. [9], who added 20 g/kg urea in FTMR for dairy cows, and Xu et al. [28], who supplemented urea at 30 g/kg DM in fattening lambs. The addition of urea had no effect on serum SCN^−^. According to Supapong and Cherdthong. [9], adding 20 g/kg of urea to the FTMR feed had no impact on the serum SCN^−^ levels of dairy cows.

Sulfur had no effect on total VFA or their molar proportions. Cherdthong et al. [7] discovered a nonsignificant increase in total VFA and molar proportions with 40 g/kg sulfur in a feed block given to beef cattle. Promkot et al. [3] demonstrated that adding sulfur over 5 g/kg DM had no effect on total VFA. The synthesis of VFAs from carbohydrate fermentation is linked to microbial growth and population, although sulfur had no effect on the bacterial population in our investigation. Sulfur may improve ruminal fermentation only when the diet is sulfur-deficient, as demonstrated by Kung et al. [29]. Hegarty et al. [30] showed that sulfur supplementation dramatically enhanced DM digestion, resulting in increased VFA synthesis and bacterial population in sheep fed high sulfur diets compared to sheep on low sulfur (2.5 g/kg DM) diets. Sulfur supplementation increased DM and OM digestion but did not increase the bacterial population in this investigation. Supapong and Cherdthong [9] found that 20 g/kg sulfur supplementation raised total VFA much more than 10 g/kg sulfur supplementation, which might be attributed to improvements in molar VFA proportions, DM and OM digestibility, MCP, and EMNS with sulfur supplementation. Urea addition at 20 g/kg DM concentrate considerably enhanced ruminal total VFA but had no effect on molar VFA proportions. This meant that carbohydrate fermentation in the rumen increased, whereas urea had no effect on microbial growth [31]. The greater NH_3_-N content from urea decomposition and readily carbohydrate fermentation from fresh cassava root supplementation may have resulted in a beneficial effect on total VFA with urea addition, creating a better environment for ruminal development [32, 33]. Similarly, Khattab et al. [34] found that adding 0, 10, and 15 g/kg urea, while Supapong and Cherdthong [9] found that adding 10 and 20 g/kg urea considerably boosted total VFA synthesis.

Milk production, FCM, and milk compositional were unaffected by sulfur supplementation at 10 and 20 g/kg DM concentrate. However, milk SCN^−^ and SCC were significantly impacted. Mammalian extracellular fluids such as saliva, plasma, milk, gastric juice, the fluid that lines the epithelium, the fluid that lines the nasal passages, and tears all contain SCN^−^, a minute, acidic pseudohalide thiolate. SCN^−^ was thought to be mostly derived from foods or synthesized from HCN by mitochondrial sulfur-dependent rhodanese and cytosolic mercaptopyruvate sulfur transferase. This might explain why milk SCN^−^ levels increased when sulfur levels were increased in this study. The mean milk SCN^−^ levels for 10 and 20 g/kg sulfur supplementation were 7.34 ppm and 8.64 ppm, respectively. Supapong and Cherdthong [9] discovered a significant rise in milk SCN^−^ from 5.52 to 11.17 ppm when sulfur was raised from 10 to 20 g/kg DM in FTMR-fed dairy cows. Cherdthong et al. [7] discovered that sulfur supplementation at 1.5 and 4 g/kg DM had no effect on milk SCN^−^ concentrations ranging from 14.95 to 15.35 ppm in dairy cows, despite the fact that SCN^−^ transits into milk seems to be gradual. When sulfur was raised from 10 to 20 g/kg DM of concentrate, the SCC fell from 297.81 × 10^3^ to 114.5 × 10^3^ cells/ml. The decrease in SCC may be related to an increase in milk SCN^−^ when sulfur supplementation is increased. According to certain reports, SCN^−^ possesses antibacterial qualities that might affect somatic germs. HOSCN is the end product of the reaction that occurs when SCN^−^ joins peroxidase in the presence of H_2_O_2_. HOSCN's antimicrobial action is defined by its capacity to penetrate the cell wall of bacteria, with Ellman's reagent (2-nitro-5-thiobenzoate, TNB) or an acidic cysteine moiety coupled with an enzyme as targets. Similarly, Supapong and Cherdthong [9] discovered that increasing sulfur to 20 g/kg DM reduced SCC in dairy cows given FTMR. As a result, milk SCN^−^ with antibacterial capabilities may be useful in extending milk shelf-life. Urea had no effect on milk output, FCM, milk composition, milk SCN^−^, or SCC, which might be attributed to the meals' identical nutritional content [35]. Dietary coated urea had no impact on the production or composition of milk, according to Xin et al. [36]. Similar to this, Khattab et al. [34] showed that the use of slow-release urea had no effect on the volume or composition of milk. Supapong and Cherdthong [9] found that the addition of 20 g/kg urea dramatically increased milk fat and total solids, which was likely due to better DM, OM, and CP digestion, MCP, and altered molar proportions of VFA, notably acetate and butyrate.

## 6. Conclusion

This study showed that the addition of sulfur and urea had no interaction effect on feed utilization, rumen fermentation, or milk production. In comparison with sulfur at a concentration of 10 g/kg in a concentrate diet, sulfur at 20 g/kg increased milk and serum SCN^−^ and lowered SCC while also improving DM and OM digestibility. When compared to concentrate diet urea levels of 10 g/kg, 20 g/kg significantly enhanced CP digestibility, NH_3_-N concentration, BUN, and total VFA. We suggested that urea and sulfur may contribute up to 20 g/kg to the concentrate-based diet when fresh cassava root was supplemented at a rate of 15 g DM/kg of BW.

## Figures and Tables

**Table 1:** Ingredient and chemical composition of concentrate, cassava roots, and rice straw.

	Sulfur-1		Sulfur-2		Fresh cassava root	Rice straw
	Urea-1	Urea-2	Urea-1	Urea-2		
Ingredients, g/kg DM						
Cassava chip	550	540	540	530		
Rice bran	130	130	130	130		
Soybean meal	230	230	230	230		
Palm kernel meal	40	40	40	40		
Minerals and vitamin	10	10	10	10		
Molasses	10	10	10	10		
Salt	10	10	10	10		
Sulfur	10	10	20	20		
Urea	10	20	10	20		

Chemical composition						
Dry matter, g/kg	878.0	882.0	877.0	880.0	332.0	946.0
Organic matter, g/kg DM	927.0	925.0	933.0	931.0	985.0	932.0
Crude protein, g/kg DM	163.6	185.3	162.0	186.4	24.0	26.0
NDF, g/kg DM	123.4	124.1	123.0	124.3	530.0	665.0
ADF, g/kg DM	84.0	84.6	84.3	84.4	314.0	434.0
HCN, g/kg DM					0.105	

Sulfur-1 and sulfur-2 = sulfur addition into concentrate at 10 g/kg DM and 20 g/kg DM. Urea-1 and urea-2 = urea addition into concentrate at 10 g/kg DM and 20 g/kg DM; DM = dry matter; NDF = neutral detergent fiber; ADF = acid detergent fiber; HCN = hydrogen cyanide.

**Table 2:** Effect of sulfur and urea levels on feed intake and nutrient digestibility in dairy cows.

Items	Sulfur-1		Sulfur-2		SEM	*P* value		
	Urea-1	Urea-2	Urea-1	Urea-2		Sulfur	Urea	Sulfur ^*∗*^ urea
RS intake								
kg DM/d	3.75	3.85	3.82	3.87	0.035	0.488	0.305	0.727
% BW	0.83	0.85	0.85	0.86	0.007	0.412	0.222	0.683
g/kg BW^0.75^	38.25	39.28	39.02	39.54	0.277	0.372	0.186	0.655

Concentrate intake								
kg DM/d	5.62	5.62	5.62	5.62	0.130	1.000	1.000	1.000
% BW	1.24	1.24	1.24	1.24	0.024	0.979	0.996	0.985
g/kg BW^0.75^	57.34	57.32	57.36	57.40	1.139	0.982	0.997	0.989
Fresh cassava root intake, kg DM/d	6.82	6.82	6.82	6.82	0.062	1.000	1.000	1.000
HCN intake, mg/d	713.89	713.89	713.89	713.89	6.537	1.000	1.000	1.000
Urea intake, g/d	56.25	56.25	112.50	112.50	1.960	<.0001	1.000	1.000

Sulfur intake								
g/d	56.25	56.25	112.50	112.50	1.960	<.0001	1.000	1.000

Total DM intake								
kg DM/d	16.20	16.30	16.27	16.32	0.197	0.901	0.852	0.950
% BW	3.58	3.60	3.60	3.61	0.026	0.811	0.746	0.931
g/kg BW^0.75^	165.21	166.22	166.00	166.56	1.283	0.829	0.765	0.932

Digestibility								
DM, g/kg	652.3^a^	662.1^a^	685.1^b^	686.3^b^	1.02	0.013	0.078	0.064
OM, g/kg DM	698.4^a^	700.5^a^	712.4^b^	715.6^b^	1.12	0.034	0.094	0.087
CP, g/kg DM	616.5^a^	621.7^b^	613.7^a^	631.3^b^	0.160	0.309	0.004	0.076
NDF, g/kg DM	594.2	596.1	598.0	596.4	2.40	0.094	0.085	0.095
ADF, g/kg DM	385.4	392.5	394.5	387.6	1.58	0.092	0.124	0.093

Sulfur-1 and sulfur-2 = sulfur addition into concentrates at 10 g/kg DM and 20 g/kg DM. Urea-1 and urea-2 = urea addition into concentrates at 10 g/kg DM and 20 g/kg DM; SEM = standard error of mean; BW = body weight; BW^0.75^ = metabolic body weight; DM = dry matter; OM = organic matter; CP = crude protein; NDF = neutral detergent fiber; ADF = acid detergent fiber; sulfur ^*∗*^ urea = interaction effect between sulfur and urea; ^a, b^means in the same row with different superscript letters are accepted as significantly different (*p* < 0.05).

**Table 3:** Effect of sulfur and urea levels on rumen ecology in dairy cows.

Items	Sulfur-1		Sulfur-2		SEM	*P* value		
	Urea-1	Urea-2	Urea-1	Urea-2		Sulfur	Urea	Sulfur ^*∗*^ urea
Ruminal pH								
0 h prefeeding	6.65	6.67	6.67	6.57	0.071	0.606	0.582	0.376
4 h postfeeding	6.47	6.41	6.39	6.32	0.094	0.064	0.474	0.896
Mean	6.56	6.54	6.53	6.44	0.047	0.244	0.306	0.495

Ammonia-nitrogen, mg/dl								
0 h prefeeding	8.51^a^	10.60^b^	8.92^a^	11.52^b^	0.128	0.151	0.001	0.108
4 h postfeeding	18.61^a^	23.61^b^	19.22^a^	24.11^b^	0.156	0.165	0.001	0.964
Mean	13.56^a^	17.14^b^	14.09^a^	17.85^b^	0.118	0.189	0.001	0.432

Protozoa, ×10^6^ cells/mL								
0 h prefeeding	8.80	9.00	8.90	8.80	0.081	0.457	0.880	0.117
4 h postfeeding	12.80	12.90	12.80	12.90	0.049	0.805	0.231	0.463
Mean	10.83	10.95	10.87	9.31	0.787	0.329	0.375	0.308

Bacteria, ×10^9^ cells/mL								
0 h prefeeding	31.00	31.00	30.50	30.50	0.549	0.381	1.000	1.000
4 h postfeeding	41.75^a^	41.25^a^	41.50^a^	42.25^b^	0.295	0.164	0.836	0.038
Mean	36.38	36.06	36.00	36.38	0.354	0.931	0.931	0.352

Sulfur-1 and sulfur-2 = sulfur addition into concentrates at 10 g/kg DM and 20 g/kg DM. Urea-1 and urea-2 = urea addition into concentrates at 10 g/kg DM and 20 g/kg DM; SEM = standard error of mean; sulfur ^*∗*^ urea = interaction effect between sulfur and urea; ^a,b^means in the same row with different superscript letters are accepted as significantly different (*p* < 0.05).

**Table 4:** Effect of sulfur and urea levels on thiocyanate (SCN^−^), blood urea nitrogen (BUN), thyroid hormones, and liver enzymes in dairy cows.

Items	Sulfur-1		Sulfur-2		SEM	*P* value		
	Urea-1	Urea-2	Urea-1	Urea-2		Sulfur	Urea	Sulfur ^*∗*^ urea
Serum SCN^−^, *μ*g/ml	13.75^a^	13.25^a^	16.25^b^	16.00^b^	0.86	0.011	0.673	0.888
BUN, mg/dl	8.85^a^	9.35^b^	8.94^a^	9.83^b^	0.14	0.097	0.001	0.064
T3, mg/dl	165.48	165.73	164.05	164.65	8.39	0.884	0.961	0.984
T4, mg/dl	8.04	7.26	8.29	7.25	1.32	0.930	0.504	0.921
ALT, *µ*/l	24.25	22.50	26.75	22.00	2.71	0.719	0.254	0.591
AST, *µ*/l	81.50	80.50	82.25	81.75	6.16	0.873	0.905	0.968

Sulfur-1 and sulfur-2 = sulfur addition into concentrate at 10 g/kg DM and 20 g/kg DM. Urea-1 and urea-2 = urea addition into concentrate at 10 g/kg DM and 20 g/kg DM; SEM = standard error of mean; T3 = triiodothyronine; ALT = alanine aminotransferase; ASTS = aspartate aminotransferase (AST); T4 = thyroxine; sulfur ^*∗*^ urea = interaction effect between sulfur and urea; ^a,b^means in the same row with different superscript letters are accepted as significantly different (*p* < 0.05).

**Table 5:** Effect of sulfur and urea levels on total VFA and its molar proportions in dairy cows.

Item	Sulfur-1		Sulfur-2		SEM	*P* value		
	Urea-1	Urea-2	Urea-1	Urea-2		Sulfur	Urea	Sulfur ^*∗*^ urea
Total VFA, mM								
0 h prefeeding	107.10	106.90	106.90	106.80	0.23	0.259	0.406	0.406
4 h postfeeding	109.10^a^	129.30^b^	106.10^a^	131.00^b^	0.45	0.417	0.001	0.784

Acetic acid, mole/100 moles								
0 h prefeeding	64.10	64.20	64.60	64.30	0.24	0.230	0.757	0.475
4 h postfeeding	62.20	62.20	62.10	62.00	0.27	0.563	0.754	0.893

Propionic acid, mole/100 moles								
0 h prefeeding	25.00	25.20	25.10	25.30	0.34	0.746	0.592	0.971
4 h postfeeding	27.30	27.40	28.40	28.30	0.46	0.050	0.957	0.790

Butyric acid, mole/100 moles								
0 h prefeeding	10.90	10.60	10.30	10.40	0.24	0.113	0.649	0.542
4 h postfeeding	10.50	10.50	10.50	11.80	0.42	0.069	0.884	0.706

Sulfur-1 and sulfur-2 = sulfur addition into concentrate at 10 g/kg DM and 20 g/kg DM. Urea-1 and urea-2 = urea addition into concentrate at 10 g/kg DM and 20 g/kg DM; SEM = standard error of mean; VFA = volatile fatty acid; sulfur ^*∗*^ urea = interaction effect between sulfur and urea; ^a,b^means in the same row with different superscript letters are accepted as significantly different (*p* < 0.05).

**Table 6:** Effect of sulfur and urea levels on milk yield and composition in dairy cows.

Items	Sulfur-1		Sulfur-2		SEM	*P* value		
	Urea-1	Urea-2	Urea-1	Urea-2		Sulfur	Urea	Sulfur *∗* urea
Milk yield, kg/d	10.78	10.82	10.92	10.91	0.48	0.814	0.969	0.966
3.5% FCM^*∗*^, kg/d	11.00	10.97	10.82	11.43	0.45	0.761	0.538	0.494
Fat, g/kg	36.20	35.80	35.80	36.50	0.09	0.867	0.867	0.618
Protein, g/kg	35.10	35.40	35.30	34.20	0.09	0.614	0.649	0.467
Lactose, g/kg	47.50	47.20	46.90	45.90	0.04	0.051	0.125	0.426
Solid-not-fat, g/kg	91.40	93.60	94.40	95.20	0.12	0.084	0.229	0.554
Total solids, g/kg	122.50	123.40	123.10	122.80	0.21	0.976	0.901	0.781
Milk SCN^−^, ppm	7.45^a^	7.23^a^	8.65^b^	8.63^b^	0.24	0.0001	0.610	0.682
SCC, × 10^3^, cells/ml	314.87^a^	280.75^a^	246.50^b^	211.50^b^	17.23	0.001	0.067	0.983

Sulfur-1 and sulfur-2 = sulfur addition into concentrate at 10 g/kg DM and 20 g/kg DM. Urea-1 and urea-2 = urea addition into concentrate at 10 g/kg DM and 20 g/kg DM; SEM = standard error of mean; ^*∗*^FCM = fat corrected milk (3.5% FCM = 0.432 + (0.1625 × milk fat) × milk yield); SCC = somatic cell count; sulfur ^*∗*^ urea = interaction effect between sulfur and urea; ^a,b^means in the same row with different superscript letter are accepted as significantly different (*p* < 0.05).

## Data Availability

The data that support the findings of this study are available from the corresponding author upon reasonable request.
